# Assessment of Binge-Like Eating of Unsweetened vs. Sweetened Chow Pellets in BALB/c Substrains

**DOI:** 10.3389/fnbeh.2022.944890

**Published:** 2022-07-15

**Authors:** Katherine D. Sena, Jacob A. Beierle, Kayla T. Richardson, Kathleen M. Kantak, Camron D. Bryant

**Affiliations:** ^1^Laboratory of Addiction Genetics, Department of Pharmacology and Experimental Therapeutics, Boston University School of Medicine, Boston, MA, United States; ^2^Department of Psychological and Brain Sciences, Boston University, Boston, MA, United States

**Keywords:** disordered eating, rodents, sugar, substrains, reduced complexity cross, quantitative trait

## Abstract

Binge eating disorder (BED) is defined as chronic episodes of consuming large amounts of food in less than 2 h. Binge eating disorder poses a serious public health problem, as it increases the risk of obesity, type II diabetes, and heart disease. Binge eating is a highly heritable trait; however, its genetic basis remains largely unexplored. We employed a mouse model for binge eating that focused on identifying heritable differences between inbred substrains in acute and escalated intake of sucrose-sweetened palatable food vs. unsweetened chow pellets in a limited, intermittent access paradigm. In the present study, we examined two genetically similar substrains of BALB/c mice for escalation in food consumption, incubation of craving after a no-food training period, and compulsive-like food consumption in an aversive context. BALB/cJ and BALB/cByJ mice showed comparable levels of acute and escalated consumption of palatable food across training trials. Surprisingly, BALB/cByJ mice also showed binge-like eating of the unsweetened chow pellets similar to the escalation in palatable food intake of both substrains. Finally, we replicated the well-documented decrease in anxiety-like behavior in BALB/cByJ mice in the light-dark conflict test that likely contributed to greater palatable food intake than BALB/cJ in the light arena. To summarize, BALB/cByJ mice show binge-like eating in the presence and absence of sucrose. Possible explanations for the lack of selectivity in binge-like eating across diets (e.g., novelty preference, taste) are discussed.

## Introduction

Binge eating disorder (BED) is a psychiatric disorder in which individuals exhibit a loss of control over eating (Colles et al., 2008) and consume an unusually large amount of food within a short period of time (referred to as a binge eating episode), typically less than 2 h (Hudson et al., 2006). According to the Diagnostic and Statistical Manual of Mental Disorders, Fifth Edition (DSM-5), BED is further defined as binge eating episodes occurring at least once a week for 3 months (American Psychiatric Association, 2013). Approximately 3.5% of women and 2.0% of men in the U.S. experienced BED in their lifetime BED is more common than anorexia nervosa and bulimia nervosa and increases the risk of obesity by three-six times those without eating disorders (Kessler et al., 2013), in addition to increased risk for type II diabetes, heart disease, high blood pressure, and high cholesterol (Hudson et al., 2007). BED can be fatal and is often accompanied by serious comorbidities, which underscores the importance of finding new therapies.

BED has been compared to Substance Use Disorders (SUDs) because both share features such as diminished control over use or consumption, continued use despite negative consequences, loss of control during use or consumption, and feelings of cravings and negative affect (Davis and Carter, 2009; Gearhardt et al., 2011). Importantly, nearly 1 in 10 BED patients have a comorbid substance abuse disorder (Ulfvebrand et al., 2015). Furthermore, binge eating activates the dopamine reward pathways (Balodis et al., 2014; Moore et al., 2018). Similar to drug or alcohol addiction, over time, BED ultimately reduces the basal activity of the reward circuitry and induces a negative affective state such that consumption of the respective substance becomes negatively reinforcing and alleviates the negative emotional state of withdrawal (Parylak et al., 2011).

Twin studies indicate that the heritability of BED ranges from 39% to 45% (Yilmaz et al., 2015; Bulik et al., 2022), and if an individual has a family member with BED, that individual is two times more likely to have BED than individuals without a family history of BED (Hudson et al., 2006). However, the genetic basis of BED is unknown. A preprint of a human genome-wide association study (GWAS) using data from the Million Veterans Program reported three risk loci near the *HFE*, *MCHR2*, and *LRP11* genes influencing binge eating (Burstein et al., 2022), and further human genome-wide polygenic scores found genetic correlations in individuals with “binge-type” eating disorders and psychiatric disorders, like ADHD and depression (Hübel et al., 2021). There is only one FDA-approved drug for BED, namely, the amphetamine-like compound lisdexamfetamine (Griffiths et al., 2021). Improving our understanding of the genetic basis of BED will potentially aid in the development of new therapeutic approaches.

Mice remain the premier mammalian model organism for understanding the genetic and molecular mechanisms of complex traits that model aspects of complex diseases and disorders, permitting control over environmental variance, diet composition, and access to diet than in human studies and providing the molecular tools for validation. We previously described genetic elements mediating differences between the C57BL/6J and DBA/2J strains and mapped quantitative trait loci on chromosomes 5 and 11 responsible for initial palatable food intake and the escalation of palatable food consumption respectively (Babbs et al., 2018; Yao et al., 2021). Large phenotypic differences between closely related substrains, such as with C57BL/6 substrains (Kirkpatrick et al., 2017), can greatly facilitate genetic mapping and gene identification in an F2 reduced complexity cross (the generation and testing of a filial generation 2 population, produced by intercrossing of substrains) by reducing the density of genetic polymorphisms, reducing the chance of gene × gene interactions, and increasing the likelihood of identifying a single causal locus (Bryant et al., 2018, 2020). We previously employed an F2 reduced complexity cross between nearly identical C57BL/6 substrains to successfully map and validate a missense mutation in cytoplasmic FMR1-interacting protein 2 *(Cyfip2)* as a causal gene underlying robust escalation in palatable food consumption in C57BL/6NJ mice compared to C57BL/6J (Kirkpatrick et al., 2017). Mice with the *Cyfip2*^N/N^ allele demonstrated greater binge-like eating (Kirkpatrick et al., 2017) and this gene is closely related to *Cyfip1* which is implicated in Prader-Willi involving hyperphagia (Angulo et al., 2015). We subsequently showed that heterozygous deletion of Cyfip1 also induced complex modulation of binge-like eating that depended on both Sex and the Cyfip2 genetic background (Babbs et al., 2018).

The BALB/cJ and BALB/cByJ mouse substrains were separated in 1935 after the 37th filial (F) generation of inbreeding (Potter, 1985). The two substrains are nearly isogenic, differing by fewer than 10,000 estimated single nucleotide polymorphisms and indels (Beierle et al., 2022b). Despite their genetic similarity, these substrains frequently show robust differences in several behaviors relevant to psychiatric disorders, including social aggression, pain sensitivity, and opioid metabolism (Michalikova et al., 2010; Velez et al., 2010; Sittig et al., 2014; Bryant et al., 2020; Beierle et al., 2022b). BALB/c substrains also express differences in food and addiction related behaviors, where BALB/cJ mice show increased acquisition of operant conditioning for a food reward, decreased extinguishing of operant responses for food, increased anxiety phenotypes, and decreased acquisition of alcohol consumption (Blizard et al., 2004; Sittig et al., 2014; Dam et al., 2019; Beierle et al., 2022a). Because of these behavioral differences, and because we successfully mapped the genetic basis of other phenotypes in BALB/c substrains (Beierle et al., 2022a, b), BALB/c substrains represent an excellent model system for the genetic analysis of complex traits and behaviors such as binge-like eating. Identification of differences in binge-like eating traits in closely related substrains, such as the BALB/c mice paves the way for rapid determination of underlying causal genes (Bryant et al., 2018, 2020) that could have translational genetic relevance to BED (Bulik et al., 2022).

In this study, we examined binge-like eating of sweetened palatable food vs. control chow pellets in BALB/c substrains. We hypothesized that we would observe increased escalation and magnitude of binge-like eating of sweetened food in BALB/cJ mice, with no substrain difference in consumption in the chow groups. We based this hypothesis on a previous report showing increased acquisition and delayed extinction in operant food paradigms between BALB/cJ and BALB/cByJ mice (Dam et al., 2019). To test our hypothesis, we employed a model of limited intermittent access to sucrose-sweetened palatable food vs. control chow pellets, without any stressor exposure or food restriction. We expected escalation of the palatable food, but not chow, a commonly observed phenomenon in this model (Boggiano et al., 2007; Corwin et al., 2011; Lardeux et al., 2013; Kirkpatrick et al., 2017; Babbs et al., 2018). Additionally, we measured related behavioral correlates of anxiety (locomotion), neophobia (bowl approach), incubation of craving for palatable food after 1 week of no access (Grimm et al., 2005; Darling et al., 2016), the persistence of investigating the food bowl during this no food training period, and a light/dark conflict test to measure the drive to consume palatable food in the face of aversive stimuli in order to assess these phenotypes alongside consumption (Kronenberger and Médioni, 1985; Lardeux et al., 2013).

## Materials and Methods

### Mice

All experiments were performed in accordance with the National Institutes of Health Guidelines for the Use of Laboratory Animals and were approved by the Institutional Animal Care and Use Committee at Boston University (National Research Council (US) Committee for the Update of the Guide for the Care and Use of Laboratory Animals, 2011). Adult BALB/cJ (J) and BALB/cByJ(ByJ) mice (7 weeks old) were purchased from the Jackson Laboratory (Bar Harbor, ME; #000651, #001026), housed four per cage and allowed one week of habituation in the vivarium before testing began. Mice were maintained on Teklad diet (Envigo, Indiana; #2018; contains 3.1 kcal/g: 24% from protein, 18% from fat, and 58% from carbohydrates) and a 12 h light/dark cycle (lights on 06:30–18:30). All testing began at 14:00. Two cohorts of mice (32 per cohort at day 1; each cohort was equally balanced by sex, substrain, and treatment) were tested over a 3-month time period. Across both cohorts, we tested 16 mice per substrain and diet group (eight M, eight F). Two male palatable food ByJ mice were euthanized following testing on day 29 due to fight wounds, and were not tested in the light-dark apparatus. These two mice have been included in all food consumption analyses but excluded from the light-dark analysis. An additional male palatable food J mouse was euthanized following testing on day 10 because of being singly housed due to fighting, which has been excluded from all analysis. Sample sizes for each analysis are provided in figure legends.

### Food Pellets

Chow and palatable food pellets were acquired from TestDiet (Richmond, IN, USA). Palatable food pellets (PF; TestDiet #1811142, 5TUL, LabTab AIN-76A Rodent 20 mg) contain 3.44 kcal/g (20.6% from protein, 12.7% from fat, and 66.7% from carbohydrates). Chow pellets (CH; TestDiet #1816662-371, 5BR3, 5V75 Tablet 20mg) were designed by Purina LabDiet to closely resemble the home cage diet (Teklad 18% Protein Diet). Chow pellets are 3.26 kcal/g (calories provided: 23% from protein, 13% from fat, and 64% from carbohydrates). Further information for the 5TUL pellets can be accessed on the TestDiet website^1^. Additionally, more information regarding the 5V75 pellets is available on the LabDiet website^2^.

### Binge Eating Training and Re-Exposure

**Figure 1:**
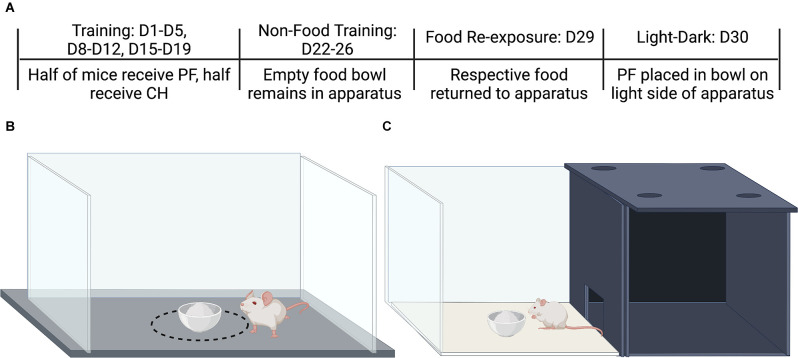
Protocol for food intake training trials, no-food training, re-exposure of food availability, and the light-dark conflict test for compulsive-like food intake. **(A)** Testing schedule for assessing acute and escalated food intake. On training days (D1-D5; D8-D12; D15-D19), mice of each Substrain and Sex received either palatable food **(PF)** or Chow **(CH)**. During the no-food training period (D22-D26), the food bowl remained in the apparatus but no food was provided. On the day of re-exposure of food availability (D29), the food was returned to the apparatus. The day following re-exposure (D30), mice were placed on the dark side with open access to the light side which contained a food bowl filled with palatable food for all groups, including chow-trained groups. Cartoons were made using BioRender.com. **(B)** Apparatus used for training, no-food training, and re-exposure. The dashed black circle around the food bowl (14.4 cm in diameter) indicates the area used to estimate the “Circle time.” **(C)** Light-dark conflict testing apparatus. Figure made using BioRender.

BALB/c substrains were tested for escalation of food intake under non-food deprived conditions as a model of binge-like eating using a 30-day paradigm containing four phases: binge-like escalation, no-food training period, food re-exposure, and light-dark conflict test (Figure 1A). For binge-like escalation, no-food training period, and re-exposure, single mice were placed in dark, sound attenuating chambers containing a Plexiglas box (21.6 cm ×43.2 cm) with a food bowl in the center of the apparatus (Figure 1B). Between testing, the apparatuses were cleaned with 20% ethanol. Mice were weighed on all testing days prior to being placed in the apparatuses. During training days 1–5, 8–12, and 15–19, half of the mice were given access to palatable food in the food bowl for 30 min, and the other half were provided with access to chow. All testing sessions lasted 30 min, and mice were provided an ad libitum amount of 40–50 pellets (none of the mice ever finished all pellets) during binge escalation and re-exposure. Food consumption was measured by determining the weight difference prior to and following testing, where all spillage was collected from the apparatus. During the no-food training period, on days 22–26, mice were placed into the apparatus for 30 min, with clean, empty food bowls. Finally, we reinstated the availability of food on day 29, mice were once again given access to their respective training diet: either palatable food or chow. The no-food training period followed by re-exposure attempted to mimic diet and relapse patterns in humans where exposure to food and/or food-associated cues after a period of no-food drives increasing consumption (Nederkoorn and Jansen, 2002). This is observed in rodent models, wherein removal and subsequent reinstatement of sucrose access drives increased consumption (Grimm et al., 2005; Darling et al., 2016). Total food consumption, distance traveled, and time near the food bowl were recorded during testing. Locomotion (distance traveled) was used as a measure of anxiety. Time near the food bowl was measured to determine if mice experienced neophobia with the food introduced in this context (Kronenberger and Médioni, 1985) and to determine if mice consumed their food near the food bowl or away from the food bowl (Babbs et al., 2018). No testing occurred on days 6, 7, 13, 14, 20, and 21. Mice were always provided with unrestricted access to home-cage food outside of the 30 min trials. Changes in body weight were assessed as percent changes from day 1.

### Light-Dark Conflict Test

The light-dark conflict test was run on D30, at the same time as training and re-exposure testing, as a measure of compulsive-like eating in the presence of aversive stimuli (i.e., light; Kirkpatrick et al., 2017; Moore et al., 2018). The apparatus consisted of one side made of black opaque Plexiglas and a second side transparent and light exposed, with a small doorway allowing access to both sides (40 cm × 40 cm; Figure 1C). For all mice, the center of the light side contained a porcelain bowl containing palatable food. At the start of testing, each mouse was placed on the dark side and recorded for 30 min. Time spent on the light side (s), amount of palatable food consumed (g), and the number of entries were reported. In contrast to the re-exposure test on D29 where mice received their respective training diet, for D30 in the light-dark test, all mice were presented with palatable food (i.e., for the chow-trained mice, palatable food is a novel food stimulus).

### Behavioral Analysis

All behavioral videos were tracked using ANY-maze (Wood Dale, IL) and subsequent statistical analysis was conducted in R^3^ using multi-factor ANOVAs considering Diet, Sex, and Substrain, and repeated measures ANOVAs considering Diet, Sex, Substrain, and Day as described in the results, with an alpha level of 0.05 to detect main effects and interactions. *Post-hoc* analysis was performed using Tukey’s HSD test (*p* < 0.05). All error bars represent the standard error of the mean (SEM). Cohen’s F was calculated to determine the effect size of the results.

## Results

### Effect of Substrain and Diet on Food Consumption

The binge eating protocol and apparatus are pictured in Figures 1A,B. The dashed line in Figure 1B represents the “circle” to measure the time the animals spent adjacent to the food bowl and was 14.4 cm in diameter. The light-dark apparatus used for assessing compulsive-like eating is shown in Figure 1C.

**Figure 2:**
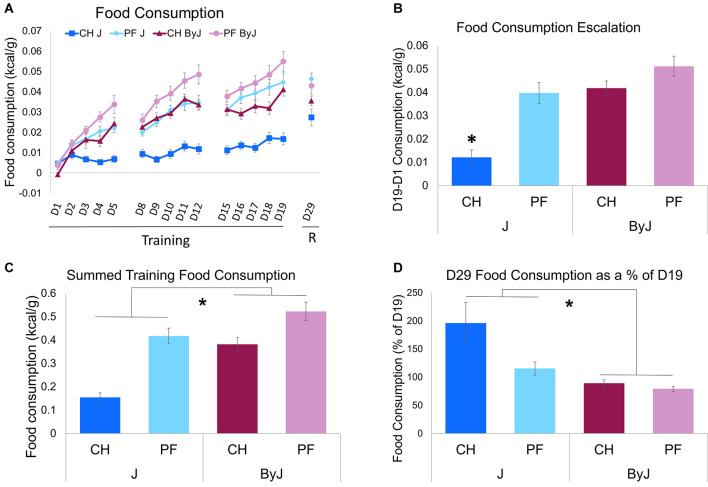
Acute and escalated Palatable Food (PF) intake vs. Chow (CH) intake in BALB/cJ and BALB/cByJ substrains. **(A)** Food consumption across training days and during the re-exposure trial “R”. **(B)** Escalation of food consumption over training, normalized to body weight (D19-D1; * interaction between Diet and Substrain (*F*_(1, 55)_ = 6.14, *p = 0.016). **(C)** Summation of the total food consumed during training trials; * main effect of Substrain (*F*_(1, 55)_ = 30.80, *p = 8.5e-7). **(D)** Change in food consumption from training to re-exposure. As a measure of re-exposure, the change in consumption (g/kcal) was examined from the final day of training (D19) to re-exposure (D29; * main effect of Substrain (*F*_(1, 55)_ = 12.69, *p = 0.00077). Ns = 16 J female (8 PF, 8 CH), 15 J male (7 PF, 8 CH), 16 ByJ female (8 PF, 8 CH), 16 ByJ Male (8 PF, 8 CH).

The consumption separated by Diet and Substrain across all training days can be seen in Figure 2A. To assess food consumption over training, we ran repeated measures ANOVA considering the effect of Day, Substrain, and Diet on food consumption normalized to body weight (kcal/g). We observed a main effect of Day (*F*_(14,810)_ = 1.93, *p* = 0.021; Cohen’s *f* = 1.30), an interaction between Day and Substrain (*F*_(14,810)_ = 4.33, *p* = 1.8e-7; Cohen’s *f* = 0.40) and an interaction between Day and Diet (*F*_(14,810)_ = 4.50, *p* = 7.4e-8; Cohen’s *f* = 0.39; Figure 2A). We also observed an interaction between Substrain and Sex (*F*_(1,810)_ = 5.97, *p* = 0.015; Cohen’s *f* = 0.12) and Diet and Sex (*F*_(1,810)_ = 7.46, *p* = 0.0065; Cohen’s *f* = 0.13). No three-way interaction between Day, Substrain, and Diet was observed (*p* = 0.56), nor a three-way interaction between Substrain, Diet, and Sex (*p* = 0.44). However, we observed an interaction between Diet and Substrain (*F*_(1,810)_ = 35.28, *p* = 4.2e-9; Cohen’s *f* = 0.26).

To assess for escalation of food consumption over training, body weight normalized consumption on D1 food consumption was subtracted from D19 (the final day of training) food consumption (kcal/g). Analysis of food consumption escalation revealed a main effect of Substrain (*F*_(155)_ = 32.95, *p* = 4.2e-7; Cohen’s *f* = 0.78), Diet (*F*_(155)_ = 26.80, *p* = 3.3e-6; Cohen’s *f* = 0.68), and Sex (*F*_(155)_ = 8.06, *p* = 0.0063; Cohen’s *f* = 0.38), as well as an interaction between Substrain and Diet (*F*_(155)_ = 6.14, *p* = 0.016; Cohen’s *f* = 0.33). Tukey’s * post-hoc* tests revealed significantly greater escalation of food consumption in the chow ByJ, palatable food ByJ, and palatable food J groups vs. chow J group (*p < 0.0001; Figure 2B). The escalation of unsweetened chow pellet intake in ByJ was not significantly different from either the palatable food ByJ group or the palatable food J group (*p* = 0.26; Figure 2B).

Analysis of summed food consumption across training days using a three-way ANOVA considering Diet, Substrain, and Sex revealed a main effect of Diet (*F*_(155)_ = 45.47, *p* = 9.7e-9; Cohen’s *f* = 0.89), Substrain (*F*_(155)_ = 30.80, *p* = 8.5e-7; Cohen’s *f* = 0.75), and Sex (*F*_(155)_ = 6.69, *p* = 0.012; Cohen’s *F* = 0.35). The interaction between Diet and Substrain was nearly significant (*p* = 0.052; Figure 2C).

In examining the percent change in consumption between last day of training (D19) and reintroduction of food (D29) as a measure of incubation of intake [D29 (g consumed)/D19 (g consumed)] * 100, three-way ANOVA (Diet, Substrain, and Sex), revealed a main effect of Diet (*F*_(155)_ = 5.14, *p* = 0.027; Cohen’s *f* = 0.30). There was also a main effect of Substrain (*F*_(155)_ = 12.69, *p = 0.00077; Cohen’s *f* = 0.48; Figure 2D). The interaction between Diet and Substrain was not significant (*p* = 0.086). Thus, following no-food training, chow pellets reinstated food consumption to a relatively greater extent than palatable food pellets overall, and J mice reinstated food consumption to a relatively greater extent than ByJ mice overall. These findings suggest that escalation of palatable food pellets was maximally expressed at the last day of palatable food intake regardless of substrain and that J mice were more likely to escalate food consumption than ByJ mice in general following the no-food training period.

In addition to the food consumption analyses, we conducted analysis of behavioral activity measures during training, no-food training period, and re-exposure to food availability. We examined the amount of time spent near the food bowl (Circle), indicated by the dashed line around the food bowl in Figure 1B, and the total distance traveled. These behavioral assays were largely negative and in no cases were there any significant Diet × Substrain interactions (data not shown). The mean total distance (m) for each group was not significantly different on overall training days (CH J: 55.09 ± 1.76; PF J: 63.51 ± 2.06; CH ByJ: 56.61 ± 2.25; PF ByJ: 61.05 ± 2.10). The mean circle time (seconds) for each group was not significantly different on overall training days (CH J: 712.28 ± 23.21; PF J: 702.62 ± 21.76; CH ByJ:742.26 ± 16.91; PF ByJ: 690.36 ± 16.93).

### Change in Body Weight During Training and No-Food Training Period

**Figure 3:**
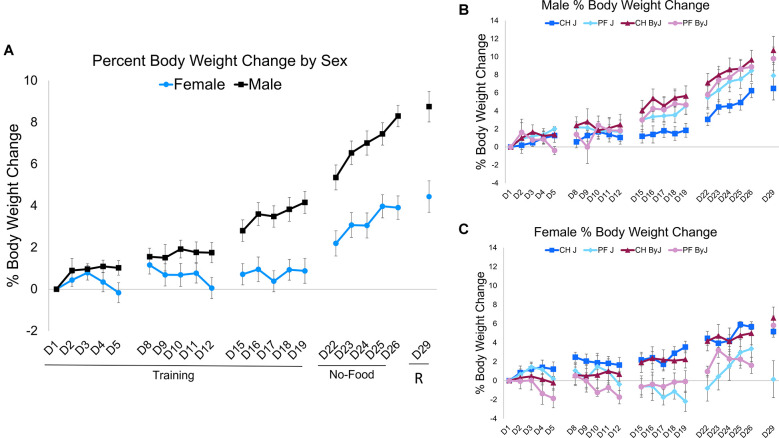
Changes in body weight during food intake trials (D1-D19) and during no-food training/re-exposure trials (D22-D29). **(A)** Body weight across training trials, no-food training, and re-exposure of food intake. Percent change in body weight from day 1. For the Training period, repeated measures (Day) ANOVA (Diet, Substrain, Sex) indicated no main effect of Day (*F*_(13,763)_ = 1.52, *p* = 0.10). However, there was an interaction between Day andSubstrain (*F*_(13,763)_ = 2.15, *p* = 0.010) and an interaction between Day and Sex (*F*_(13,763)_ = 2.88, *p* = 0.00045). In considering percent body weight change during the no-food training and re-exposure period, repeated measures (Day) ANOVA (Diet, Substrain, Sex) indicated no main effect of Day (*F*_(5,324)_ = 0.37, *p* = 0.87) nor were there any significant interactions (*p*s > 0.44). **(B)** Male body weight across training trials. **(C)** Female body weight across training trials. Ns = 16 J female (8 PF, 8 CH), 15 J male (7 PF, 8 CH), 16 ByJ female (8 PF, 8 CH), 16 ByJ Male (8 PF, 8 CH).

Because of different diet conditions during the Training period (D1-D19) vs. the no-food training/re-exposure period (D22-D29), separate repeated measures ANOVAs were conducted for each phase to assess changes in body weight (Figure 3A). For the Training period, repeated measures (Day) ANOVA (Diet, Substrain, Sex) indicated no main effect of Day (*p* = 0.10). However, there was a main effect of Sex (*F*_(1,763)_ = 21.90, *p* = 0.010; Cohen’s *f* = 0.41) and an interaction between Day and Sex (*F*_(13, 763)_ = 2.88, *p* = 0.00045; Cohen’s *f* = 0.33) and an interaction between Day and Substrain (*F*_(13, 763)_ = 2.15, *p* = 0.010; Cohen’s *f* = 0.33). Tukey’s *post-hoc* testing of the Day × Sex interaction revealed that male mice had a greater percent increase in body weight than female mice in the final 6 days of training (*p* < 0.018; Figures 3B,C). No significant three-way interactions were observed between Day, Substrain, and Diet (*p* = 0.99) or between Day, Substrain, and Sex (*p* = 0.99).

In considering percent body weight change during the no-food training and Re-exposure period, repeated measures (Day) ANOVA (Diet, Substrain, Sex) indicated no main effect of Day (*p* = 0.87) nor was there a significant interaction between Substrain and Sex (*p* = 0.29). However, there was a main effect of Sex (*F*_(1, 324)_ = 156.26, *p* < 2e-16; Cohen’s *f* = 0.72) as well as an interaction between Substrain and Sex (*F*_(1, 324)_ = 7.54, *p* = 0.0064; Cohen’s *f* = 0.16) and Diet and Sex (*F*_(1, 324)_ = 32.35, *p* = 2.9e-8; Cohen’s *f* = 0.34). Despite the differences in testing protocol during reinstatement (D29), these body weight measurements have been included in the repeated measures ANOVA because mice were weighed before the trial took place, i.e., before re-exposure to testing diet (just like the first day of any of the other 3 weeks, namely D1, D8, D15, and D22).

### Light-Dark Conflict Assay of Compulsive-Like Eating

**Figure 4:**
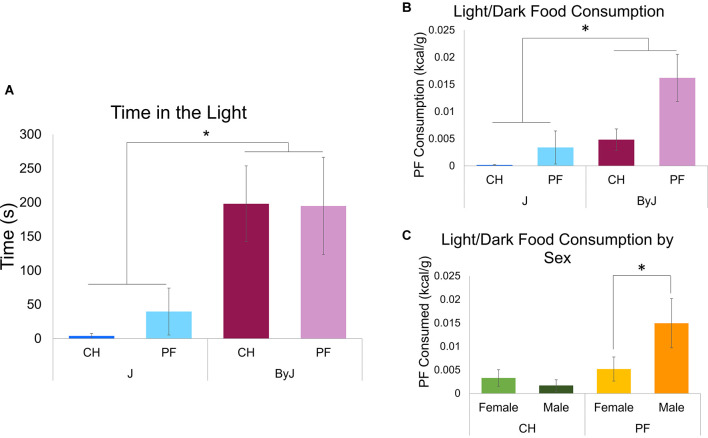
Testing for compulsive-like eating in the light-dark test on D30. **(A)** Time spent in the light side (s) while all groups were presented with a bowl filled with PF in the center of the light side (see Figure 1 for schematic). A main effect of Substrain was observed (*p = 0.00043). **(B)** Palatable food consumption during the light-dark conflict test for compulsive-like eating (main effect of Substrain:(*p = 0.0024); main effect of Diet: *p* = 0.0063). **(C)** Light dark testing palatable food consumption by Sex and Training Diet (Diet x Sex interaction: *p* = 0.016). Tukey’s *post-hoc* test revealed a significant increase in compulsive-like PF consumption in the light-dark test in the PF M vs. PF F group (*p = 0.0368). Ns = 16 J female (8 PF, 8 CH), 15 J male (7 PF, 8 CH), 16 ByJ female (8 PF, 8 CH), 14 ByJ Male (6 PF, 8 CH).

In examining the amount of time spent in the light side of the light-dark apparatus, three-way ANOVA (Diet, Substrain, and Sex), indicated a main effect of Substrain (*F*_(153)_ = 13.44, *p = 0.00057; Cohen’s *f* = 0.50; Figure 4A) but no main effect of Diet (*p* = 0.77) and no interaction between Diet and Substrain (*p* = 0.70). There was no effect of Sex (*p* = 0.23), nor were there any interactions with Sex (*p* > 0.57). Thus, ByJ mice spent a greater amount of time in the light than J mice, regardless of diet and sex.

In examining food consumption (kcal/g) during light-dark testing, three-way ANOVA (Diet, Substrain and Sex) revealed a main effect of Diet (*F*_(153)_ = 7.44, *p* = 0.0086; Cohen’s *f* = 0.42) and Substrain (*F*_(153)_ = 10.81, *p* = 0.0018; Cohen’s *f* = 0.48). Thus, there was greater consumption of palatable food pellets than chow pellets and greater food consumption by ByJ mice than J mice in the lit (more aversive) compartment of the light-dark test box (Figure 4B).

The main effect of Sex was not statistically significant (*p* = 0.14) nor was the interaction between Diet and Substrain (*p* = 0.14) or between Substrain and Sex (*p* = 0.74). Additionally, there was no significant three-way interaction between Diet, Substrain, or Sex (*p* = 0.33). However, there was an interaction between Diet and Sex (*F*_(153)_ = 5.20, *p* = 0.027; Cohen’s *f* = 0.32). Tukey’s *post-hoc* test revealed that the source of this interaction was explained by significantly greater food intake during light-dark testing in the palatable food male group vs. the palatable food female group (*p = 0.039; Figure 4C).

## Discussion

The primary finding was that while the BALB/cJ and ByJ substrains of mice both showed robust total consumption and escalation of consumption of palatable food over 15 training days, surprisingly, the ByJ substrain also binged on unsweetened chow pellets (Figure 2A). Intriguingly, despite the J substrain showing comparable escalated and summed intake of palatable food during training and less chow intake during training, when food access was reinstated after a period of no-food training, J mice showed evidence for an incubation effect whereby they showed enhanced intake on the re-exposure trial (D29), regardless of training diet (Figure 2D).

It is striking that the ByJ mice escalated their consumption of the unsweetened chow diet at a level comparable to palatable food in mice of either substrain (Figure 2B). This is the first time we have observed binge-like eating of control chow pellets in an inbred strain. Our previous studies have demonstrated binge-like consumption of palatable food compared to unsweetened chow across multiple strains (Kirkpatrick et al., 2017; Babbs et al., 2018). Interestingly, substrain differences in consumption were not accompanied by differences in food approach behavioral indices during training, no-food training, or re-exposure. Because J mice exhibit greater anxiety-like behaviors than ByJ in the open field test (Sittig et al., 2014), we considered the possibility that anxiety-like behavioral differences could be associated with differences in consumption. The lack of observed differences in time near the food bowl (i.e., the center of the apparatus) and overall locomotion (which could reveal avoidance or freezing; La-Vu et al., 2020) suggest that anxiety-like differences are unlikely to underlie differences in consumption. Indeed, increased binge-like eating is typically associated with increased anxiety-like behavior (Babbs et al., 2018) whereas here, we found the opposite, whereby the less anxious-like ByJ substrain consumed more (Figure 4A). Increased food intake in ByJ mice is likely due in part to the fact that the food was provided in the anxiety-provoking light side in which the ByJ substrain was more likely to reside. While sex has been demonstrated to have an effect on binge-like eating (Babbs et al., 2011; Klump et al., 2013), substrain and diet were our main focus and we did not observe interactions between sex, diet, and substrain to investigate. Finally, while we did observe an effect of sex on change in body weight across training these results are likely not a result of the intermittent, limited access to either palatable food or chow provided during testing, as male mice continue to gain weight longer than females, and mice received unmonitored ad libitum access to home cage food outside of testing.

One possible explanation for increased binge-like chow intake could be that ByJ mice are more impulsive-like, which could contribute to increased consumption of food independent of the rewarding value of the food. BALB/c mice have been suggested to be more “impulsive” than other inbred strains of mice (Otobe and Makino, 2004), but a direct assessment of impulsivity traits between the two substrains is yet to be explored. Impulsivity has been correlated with substance use disorders and eating disorders (Alcaraz-Iborra and Cubero, 2015; Kessler et al., 2016), particularly in the initiation of addiction behaviors, and has been demonstrated to have genetic components that have been identified in human GWAS (Dawe and Loxton, 2004; Bevilacqua and Goldman, 2013; Salatino-Oliveira et al., 2015; Giorgi et al., 2019; Sanchez-Roige et al., 2022). More specifically, treatment for attention deficit hyperactivity disorder (Lisdexamfetaminedimesylate; Vyvanse), which is stated to improve impulse control, has been shown to decrease binge-like eating (McElroy et al., 2015; Guerdjikova et al., 2016; Griffiths et al., 2021).

Another possible factor that could underlie escalated chow food intake in ByJ could be an increased preference for food/taste novelty. Novelty preference in rats has been demonstrated to be related to the compulsive aspect of addiction (Belin et al., 2011), and in humans, novelty seeking is positively correlated with binge eating prevalence (Grucza et al., 2007). Additionally, it has been shown in rats that repeated exposure to novel foods is necessary to overcome hyponeophagia and observe robust preferences for novel diet (Greiner and Petrovich, 2020). If the ByJ mice have increased preference for food/taste novelty, this could explain greater consumption of unsweetened chow, as both diets are distinct and novel from their home cage food in size, taste, and context presented. While the chow treatment pellets are lacking in sucrose, these small pellets require the inclusion of binders so that the pellets remain cohesive. These food binders are novel compared to their home cage chow and could be experienced as palatable in the ByJ substrain but not the J substrain. An alternative explanation to enhanced novelty preference in the ByJ substrain is that the J substrain may simply not be able to detect the components of the unsweetened chow pellets and thus distinguish these unsweetened pellets from their home cage chow. Furthermore, the ByJ substrain may have reduced ability to detect sucrose as a distinguishing feature of the two novel diets and thus, the novelty of the binding agents relative to their home cage chow is sufficient to drive escalated intake of both diets in the ByJ substrain. Future studies will be necessary to decipher the potential contribution of novelty preference from taste perception in explaining the binge-like eating of unsweetened chow pellets in ByJ.

Re-exposure of food availability revealed a substrain difference in the food consumption resulting in a greater increase in consumption (D29) compared to their final training day (D19) in J vs. ByJ mice suggesting an effect that could indicate incubation of the appetitive properties of the novel diets. Incubation effects have been demonstrated for sucrose consumption (Grimm et al., 2005) and high fat craving (Darling et al., 2016), which demonstrates that food and/or food-associated cues can gain saliency after a period of no-food training in driving consumption. Furthermore, increased food consumption after no-food training in J vs. ByJ mice suggests that after a period of no-food training, J mice may have experienced an incubation effect, therefore, increasing their consumption following re-exposure to food availability (Figure 2D). Incubation appears to be connected to the salience of food cues (Grimm et al., 2005; Darling et al., 2016). The J substrain shows greater sensitivity than the ByJ substrain to a reward cue in the context of extinction learning of an operant behavior (Dam et al., 2019), which could explain the incubation-like effect; however, to our knowledge incubation effects on binge eating of food pellets have not been investigated.

We replicated the previously reported decrease in anxiety-like behavior in ByJ vs. J mice in the light-dark test (Velez et al., 2010; Sittig et al., 2014). The light-dark test was designed to model compulsive-like eating whereby one can test whether the motivation for food consumption overrides the propensity to avoid the averse light context (Cottone et al., 2012). Previous studies have determined that ByJ mice show decreased anxiety-like behavior compared to J mice in the open field test (Velez et al., 2010; Sittig et al., 2014). Because ByJ mice demonstrate less anxiety-like behavior than J mice, they are more likely to spend time in the light side of the apparatus and thus more likely to consume food which confounds the ability to isolate compulsive-like eating in the face of adverse environmental stimuli. On the other hand, we previously found that the binge-prone yet anxiety-prone, DBA/2J strain was nevertheless motivated enough to brave the light side to obtain the palatable food, only to immediately return to the dark side to consume it (Babbs et al., 2018).

Future directions aimed at further dissecting other behavioral and physiological traits associated with increased chow consumption in J mice would be informative as we consider pursuing the genetic basis of this BALB/c substrain difference directly through quantitative trait locus mapping. In order to test the potential contribution of novelty (e.g., binding agents, taste, texture) to chow consumption, the same paradigm could be conducted by providing the chow pellets in the present study and comparing consumption with home cage chow in the experimental environment. Additionally, there is a need to further characterize BALB/c substrain differences in impulsivity in, e.g., an impulsive action test (e.g., five-choice serial reaction time task, stop signal task, go/no-go task) or an impulsive choice test (e.g., delay-discounting task or effort discounting task; D’Amour-Horvat and Leyton, 2014). Another possible paradigm could be a two-choice feeding test with both chow and palatable food to determine if the chow eating we observed in ByJ mice could be explained by a lack of preference between chow and palatable food (Maze Engineers., 2019). In order to determine if there is a difference in novelty preference, a novelty preference test that minimizes social factors or anxiety could be used, such as a novel object recognition test (Michalikova et al., 2010; Bryant et al., 2020).

These findings establish binge-like eating of unsweetened chow in the ByJ strain compared to the J strain of BALB/c mice two closely related substrains of BALB/c mice. The cause of this increase in chow consumption in ByJ mice is unknown but could be related to differences in impulsivity, novelty preference, or taste perception. We believe that understanding the genetic differences driving food consumption in the absence of sucrose as a palatable agent could help understand the neurobiology that maintains or initiates food consumption in binge eating paradigms, and merits future investigation. While this study provides the groundwork for future studies investigating the genetic etiology of this consumption, further studies are needed to understand the behavioral traits underpinning increased unsweetened chow consumption. Identification of additional BALB/c substrain differences in behaviors associated with binge-like eating would allow for interrogation of shared genetic factors the genetic basis of escalating chow consumption in a future reduced complexity cross (Bryant et al., 2018, 2020; Bulik et al., 2022).

## Data Availability Statement

The raw data supporting the conclusions of this article will be made available by the authors, without undue reservation.

## Ethics Statement

The animal study was reviewed and approved by Institutional Animal Care and Use Committee at Boston University School of Medicine.

## Author Contributions

KS, JB, and CB participated in study design. KS and JB conducted behavioral experiments. KS, JB, and KR measured food consumption. KS performed statistical analysis. KS, JB, KK, and CB wrote or contributed to the manuscript. All authors contributed to the article and approved the submitted version.

## Funding

This research was funded by the National Institute of Health’s National Institute on Drug Abuse (U01DA050243; U01DA055299), The National Institute of General Medical Sciences (T32GM008541), and the Burroughs Welcome Fund Transformative Training Program in Addiction Science (1011479).

## Conflict of Interest

The authors declare that the research was conducted in the absence of any commercial or financial relationships that could be construed as a potential conflict of interest.

## Publisher’s Note

All claims expressed in this article are solely those of the authors and do not necessarily represent those of their affiliated organizations, or those of the publisher, the editors and the reviewers. Any product that may be evaluated in this article, or claim that may be made by its manufacturer, is not guaranteed or endorsed by the publisher.

## Footnotes

^1^ http://www.www.testdiet.com/

^2^ http://www.labdiet.com/

^3^ http://www.r-project.org/

## References

[B1] Alcaraz-Iborra M. Cubero I. (2015). Do Orexins contribute to impulsivity-driven binge consumption of rewarding stimulus and transition to drug/food dependence? Pharmacol. Biochem. Behav. 134, 31–34. 10.1016/j.pbb.2015.04.012 25931265

[B2] American Psychiatric Association (2013). “Feeding and eating disorders,” in Diagnostic And Statistical Manual Of Mental Disorders, Fifth Edition, (Washington, D.C.: American Psychiatric Publishing), 329–354.

[B3] Angulo M. A. Butler M. G. Cataletto M. E. (2015). Prader-Willi syndrome: a review of clinical, genetic and endocrine findings. J. Endocrinol. Invest. 38, 1249–1263. 10.1007/s40618-015-0312-9 26062517 PMC4630255

[B5] Babbs R. K. Kelliher J. C. Scotellaro J. L. Luttik K. P. Mulligan M. K. Bryant C. D. (2018). Genetic differences in the behavioral organization of binge eating, conditioned food reward and compulsive-like eating in C57BL/6J and DBA/2J strains. Physiol. Behav. 197, 51–66. 10.1016/j.physbeh.2018.09.013 30261172 PMC6333425

[B6] Babbs R. K. Wojnicki F. H. E. Corwin R. L. W. (2011). Effect of 2-hydroxyestradiol on binge intake in rats. Physiol. Behav. 103, 508–512. 10.1016/j.physbeh.2011.03.029 21497615 PMC3132134

[B7] Balodis I. M. Grilo C. M. Kober H. Worhunsky P. D. White M. A. Stevens M. C.. (2014). A pilot study linking reduced fronto-Striatal recruitment during reward processing to persistent bingeing following treatment for binge-eating disorder. Int. J. Eat. Disord. 47, 376–384. 10.1002/eat.22204 24729034 PMC3986812

[B8] Beierle J. A. Yao E. J. Goldstein S. I. Lynch W. B. Scotellaro J. L. Sena K. D.. (2022a). Zhx2 is a candidate gene underlying oxymorphone metabolite brain concentration associated with state-dependent oxycodone reward. bioRxiv [Preprint]. 10.1101/2022.03.18.484877 PMC9341249 35688478

[B9] Beierle J. A. Yao E. J. Goldstein S. I. Scotellaro J. L. Sena K. D. Linnertz C. A.. (2022b). Genetic basis of thermal nociceptive sensitivity and brain weight in a BALB/c reduced complexity cross. Mol. Pain 18:17448069221079540. 10.1177/17448069221079540 35088629 PMC8891926

[B10] Belin D. Berson N. Balado E. Piazza P. V. Deroche-Gamonet V. (2011). High-novelty-preference rats are predisposed to compulsive cocaine self-administration. Neuropsychopharmacology 36, 569–579. 10.1038/npp.2010.188 20980989 PMC3055686

[B11] Bevilacqua L. Goldman D. (2013). Genetics of impulsive behaviour. Philos. Trans. R. Soc. B Biol. Sci. 368:20120380. 10.1098/rstb.2012.0380 23440466 PMC3638385

[B12] Blizard D. A. Vandenbergh D. J. Jefferson A. L. Chatlos C. D. Vogler G. P. McClearn G. E. (2004). Effects of periadolescent ethanol exposure on alcohol preference in two BALB substrains. Alcohol 34, 177–185. 10.1016/j.alcohol.2004.08.007 15902911

[B13] Boggiano M. M. Artiga A. I. Pritchett C. E. Chandler-Laney P. C. Smith M. L. Eldridge A. J. (2007). High intake of palatable food predicts binge-eating independent of susceptibility to obesity: an animal model of lean vs obese binge-eating and obesity with and without binge-eating. Int. J. Obes. (Lond) 31, 1357–1367. 10.1038/sj.ijo.0803614 17372614

[B14] Bryant C. D. Ferris M. T. De Villena F. P. M. Damaj M. I. Kumar V. Mulligan M. K. (2018). “Chapter 8 - Reduced complexity cross design for behavioral genetics,” in Molecular-Genetic and Statistical Techniques for Behavioral and Neural Research, ed Gerlai R. T. (San Diego: Academic Press), 165–190.

[B15] Bryant C. D. Smith D. J. Kantak K. M. Nowak T. S. Williams R. W. Damaj M. I.. (2020). Facilitating complex trait analysis via reduced complexity crosses. Trends Genet. 36, 549–562. 10.1016/j.tig.2020.05.003 32482413 PMC7365571

[B16] Bulik C. M. Coleman J. R. I. Hardaway J. A. Breithaupt L. Watson H. J. Bryant C. D.. (2022). Genetics and neurobiology of eating disorders. Nat. Neurosci. 25, 543–554. 10.1038/s41593-022-01071-z 35524137 PMC9744360

[B17] Burstein D. Griffen T. Therrien K. Bendl J. Venkatesh S. Dong P.. (2022). Genome-wide analysis of binge-eating disorder identifies the first three risk loci and implicates iron metabolism. medRxiv [Preprint]. 10.1101/2022.04.28.22274437 PMC10947608 37550530

[B19] Colles S. L. Dixon J. B. O’Brien P. E. (2008). Loss of control is central to psychological disturbance associated with binge eating disorder. Obesity (Silver Spring) 16, 608–614. 10.1038/oby.2007.99 18239550

[B20] Corwin R. L. Avena N. M. Boggiano M. M. (2011). Feeding and reward: perspectives from three rat models of binge eating. Physiol. Behav. 104, 87–97. 10.1016/j.physbeh.2011.04.041 21549136 PMC3132131

[B21] Cottone P. Wang X. Park J. W. Valenza M. Blasio A. Kwak J.. (2012). Antagonism of sigma-1 receptors blocks compulsive-like eating. Neuropsychopharmacology 37, 2593–2604. 10.1038/npp.2012.89 22713906 PMC3473342

[B22] D’Amour-Horvat V. Leyton M. (2014). Impulsive actions and choices in laboratory animals and humans: effects of high vs. low dopamine states produced by systemic treatments given to neurologically intact subjects. Front. Behav. Neurosci. 8:432. 10.3389/fnbeh.2014.00432 25566001 PMC4274964

[B23] Dam S. A. Jager A. Oomen C. A. Buitelaar J. K. Arias-Vasquez A. Glennon J. C. (2019). Inhibitory control in BALB/c mice sub-strains during extinction learning. Eur. Neuropsychopharmacol. 29, 509–518. 10.1016/j.euroneuro.2019.02.007 30851996

[B24] Darling R. A. Dingess P. M. Schlidt K. C. Smith E. M. Brown T. E. (2016). Incubation of food craving is independent of macronutrient composition. Sci. Rep. 6:30900. 10.1038/srep30900 27485660 PMC4971517

[B25] Davis C. Carter J. C. (2009). Compulsive overeating as an addiction disorder. A review of theory and evidence. Appetite 53, 1–8. 10.1016/j.appet.2009.05.018 19500625

[B26] Dawe S. Loxton N. J. (2004). The role of impulsivity in the development of substance use and eating disorders. Neurosci. Biobehav. Rev. 28, 343–351. 10.1016/j.neubiorev.2004.03.007 15225976

[B27] Gearhardt A. N. White M. A. Potenza M. N. (2011). Binge eating disorder and food addiction. Curr. Drug Abuse Rev. 4, 201–207. 10.2174/1874473711104030201 21999695 PMC3671377

[B28] Giorgi O. Corda M. G. Fernández-Teruel A. (2019). A genetic model of impulsivity, vulnerability to drug abuse and schizophrenia-relevant symptoms with translational potential: the roman high- vs. low-avoidance rats. Front. Behav. Neurosci. 13:145. 10.3389/fnbeh.2019.00145 31333426 PMC6624787

[B29] Greiner E. M. Petrovich G. D. (2020). The effects of novelty on food consumption in male and female rats. Physiol. Behav. 223:112970. 10.1016/j.physbeh.2020.112970 32464137 PMC7358116

[B30] Griffiths K. R. Aparício L. Braund T. A. Yang J. Harvie G. Harris A.. (2021). Impulsivity and its relationship with lisdexamfetamine dimesylate treatment in binge eating disorder. Front. Psychol. 12:716010. 10.3389/fpsyg.2021.716010 34531798 PMC8439192

[B31] Grimm J. W. Fyall A. M. Osincup D. P. (2005). Incubation of sucrose craving: effects of reduced training and sucrose pre-loading. Physiol. Behav. 84, 73–79. 10.1016/j.physbeh.2004.10.011 15642609 PMC2880539

[B32] Grucza R. A. Przybeck T. R. Cloninger C. R. (2007). Prevalence and correlates of binge eating disorder in a community sample. Compr. Psychiatry 48, 124–131. 10.1016/j.comppsych.2006.08.002 17292702 PMC1924970

[B33] Guerdjikova A. I. Mori N. Casuto L. S. McElroy S. L. (2016). Novel pharmacologic treatment in acute binge eating disorder - role of lisdexamfetamine. Neuropsychiatr. Dis. Treat. 12, 833–841. 10.2147/NDT.S80881 27143885 PMC4841437

[B34] Hübel C. Abdulkadir M. Herle M. Loos R. J. F. Breen G. Bulik C. M.. (2021). One size does not fit all. Genomics differentiates among anorexia nervosa, bulimia nervosa and binge-eating disorder. Int. J. Eat. Disord. 54, 785–793. 10.1002/eat.23481 33644868 PMC8436760

[B35] Hudson J. I. Hiripi E. Pope H. G. Kessler R. C. (2007). The prevalence and correlates of eating disorders in the National Comorbidity Survey Replication. Biol. Psychiatry 61, 348–358. 10.1016/j.biopsych.2006.03.040 16815322 PMC1892232

[B36] Hudson J. I. Lalonde J. K. Berry J. M. Pindyck L. J. Bulik C. M. Crow S. J.. (2006). Binge-eating disorder as a distinct familial phenotype in obese individuals. Arch. Gen. Psychiatry 63, 313–319. 10.1001/archpsyc.63.3.313 16520437

[B37] Kessler R. C. Berglund P. A. Chiu W. T. Deitz A. C. Hudson J. I. Shahly V.. (2013). The prevalence and correlates of binge eating disorder in the WHO World Mental Health Surveys. Biol. Psychiatry 73, 904–914. 10.1016/j.biopsych.2012.11.020 23290497 PMC3628997

[B38] Kessler R. M. Hutson P. H. Herman B. K. Potenza M. N. (2016). The neurobiological basis of binge-eating disorder. Neurosci. Biobehav. Rev. 63, 223–238. 10.1016/j.neubiorev.2016.01.013 26850211

[B39] Kirkpatrick S. L. Goldberg L. R. Yazdani N. Babbs R. K. Wu J. Reed E. R.. (2017). Cytoplasmic FMR1-interacting protein 2 is a major genetic factor underlying binge eating. Biol. Psychiatry 81, 757–769. 10.1016/j.biopsych.2016.10.021 27914629 PMC5386810

[B40] Klump K. L. Racine S. Hildebrandt B. Sisk C. L. (2013). Sex differences in binge eating patterns in male and female adult rats. Int. J. Eat. Disord. 46, 729–736. 10.1002/eat.22139 23625647

[B41] Kronenberger J.-P. Médioni J. (1985). Food neophobia in wild and laboratory mice (*Mus musculus domesticus*). Behav. Process. 11, 53–59. 10.1016/0376-6357(85)90102-0 24924361

[B42] Lardeux S. Kim J. J. Nicola S. M. (2013). Intermittent access to sweet high-fat liquid induces increased palatability and motivation to consume in a rat model of binge consumption. Physiol. Behav. 114–115, 21–31. 10.1016/j.physbeh.2013.03.005 23499930 PMC3648600

[B43] La-Vu M. Tobias B. C. Schuette P. J. Adhikari A. (2020). To approach or avoid: an introductory overview of the study of anxiety using rodent assays. Front. Behav. Neurosci. 14:145. 10.3389/fnbeh.2020.00145 33005134 PMC7479238

[B44] Maze Engineers. (2019). Feeding behaviors. Maze Eng. Available online at: https://conductscience.com/maze/feeding-behaviors/. Accessed February 27, 2022.

[B45] McElroy S. L. Hudson J. I. Mitchell J. E. Wilfley D. Ferreira-Cornwell M. C. Gao J.. (2015). Efficacy and safety of lisdexamfetamine for treatment of adults with moderate to severe binge-eating disorder: a randomized clinical trial. JAMA Psychiatry 72, 235–246. 10.1001/jamapsychiatry.2014.2162 25587645

[B46] Michalikova S. van Rensburg R. Chazot P. L. Ennaceur A. (2010). Anxiety responses in Balb/c, c57 and CD-1 mice exposed to a novel open space test. Behav. Brain Res. 207, 402–417. 10.1016/j.bbr.2009.10.028 19900487

[B47] Moore C. F. Panciera J. I. Sabino V. Cottone P. (2018). Neuropharmacology of compulsive eating. Philos. Trans. R. Soc. B Biol. Sci. 373:20170024. 10.1098/rstb.2017.0024 29352024 PMC5790823

[B48] National Research Council (US) Committee for the Update of the Guide for the Care and Use of Laboratory Animals (2011). Guide for the Care and Use of Laboratory Animals, 8th ed, The National Academies Collection: Reports funded by National Institutes of Health. Washington, DC: National Academies Press (US).

[B49] Nederkoorn C. Jansen A. (2002). Cue reactivity and regulation of food intake. Eat. Behav. 3, 61–72. 10.1016/s1471-0153(01)00045-9 15001020

[B50] Otobe T. Makino J. (2004). Impulsive choice in inbred strains of mice. Behav. Process. 67, 19–26. 10.1016/j.beproc.2004.02.001 15182922

[B51] Parylak S. L. Koob G. F. Zorrilla E. P. (2011). The dark side of food addiction. Physiol. Behav. 104, 149–156. 10.1016/j.physbeh.2011.04.063 21557958 PMC3304465

[B52] Potter M. (1985). “History of the BALB/c family,” in The BALB/c Mouse: Genetics and Immunology, Current Topics in Microbiology and Immunology, ed Pottern M. (Berlin, Heidelberg: Springer), 1–5.

[B53] Salatino-Oliveira A. Genro J. P. Polanczyk G. Zeni C. Schmitz M. Kieling C.. (2015). Cadherin-13 gene is associated with hyperactive/impulsive symptoms in attention/deficit hyperactivity disorder. Am. J. Med. Genet. B Neuropsychiatr. Genet. 168, 162–169. 10.1002/ajmg.b.32293 25739828

[B54] Sanchez-Roige S. Jennings M. V. Thorpe H. H. A. Mallari J. E. van der Werf L. Bianchi S. B.. (2022). CADM2 is implicated in impulsive personality traits by genome- and phenome-wide association studies in humans, with further support from studies of Cadm2 mutant mice. medRxiv [Preprint]. 10.1101/2022.01.29.22270095 PMC10182097 37173343

[B55] Sittig L. J. Jeong C. Tixier E. Davis J. Barrios Camacho C. M. Palmer A. A. (2014). Phenotypic instability between the near isogenic substrains BALB/cJ and BALB/cByJ. Mamm. Genome 25, 564–572. 10.1007/s00335-014-9531-1 24997021 PMC4241159

[B56] Ulfvebrand S. Birgegård A. Norring C. Högdahl L. von Hausswolff-Juhlin Y. (2015). Psychiatric comorbidity in women and men with eating disorders results from a large clinical database. Psychiatry Res. 230, 294–299. 10.1016/j.psychres.2015.09.008 26416590

[B57] Velez L. Sokoloff G. Miczek K. A. Palmer A. A. Dulawa S. C. (2010). Differences in aggressive behavior and DNA copy number variants between BALB/cJ and BALB/cByJ substrains. Behav. Genet. 40, 201–210. 10.1007/s10519-009-9325-5 20033273 PMC3722062

[B58] Yao E. J. Babbs R. K. Kelliher J. C. Luttik K. P. Borrelli K. N. Damaj M. I.. (2021). Systems genetic analysis of binge-like eating in a C57BL/6J x DBA/2J-F2 cross. Genes Brain Behav. 20:e12751. [Online ahead of print] 10.1111/gbb.12751 33978997 PMC9361732

[B59] Yilmaz Z. Hardaway J. A. Bulik C. M. (2015). Genetics and epigenetics of eating disorders. Adv. Genomics Genet. 5, 131–150. 10.2147/AGG.S55776 27013903 PMC4803116

